# A Rare Case of Primary Intraosseous Mucoepidermoid Carcinoma of the Maxilla Diagnosed by MAML2 FISH Testing After Initial Misdiagnosis

**DOI:** 10.7759/cureus.94245

**Published:** 2025-10-09

**Authors:** Koki Ueda, Masayoshi Kobayashi, Kazuhiko Takeuchi

**Affiliations:** 1 Department of Otorhinolaryngology - Head and Neck Surgery, Mie University Graduate School of Medicine, Tsu, JPN

**Keywords:** diagnosis, fluorescence in situ hybridization (fish), intraosseous mucoepidermoid carcinoma, maml2 gene, maxilla

## Abstract

We report a case of primary intraosseous mucoepidermoid carcinoma of the maxilla, an exceedingly rare condition with only nine cases documented in the literature, contributing to a prolonged diagnostic timeline. A 58-year-old woman underwent surgery four years earlier for a left maxillary mass at another hospital, initially diagnosed as a calcifying epithelial odontogenic tumor. Due to residual tumor growth, she was referred to our hospital for a second surgery. Histopathological analysis and *MAML2* gene fluorescence *in situ* hybridization (FISH) testing confirmed the diagnosis of mucoepidermoid carcinoma. A retrospective review of the tissue from the initial surgery revealed the same diagnosis. This disease poses diagnostic challenges as imaging and histological findings often resemble benign odontogenic cysts. Accurate diagnosis requires awareness of this condition and the application of definitive diagnostic tools. This case highlights the importance of considering intraosseous mucoepidermoid carcinoma in the differential diagnosis of jawbone lesions and the diagnostic utility of *MAML2* FISH testing.

## Introduction

Mucoepidermoid carcinoma is a mucus-producing epithelial tumor originating from the luminal epithelium, with the majority of cases occurring in the salivary glands. Only 2-3% of these tumors develop in the jawbone, of which most arise in the mandible. Maxillary cases are particularly rare, accounting for only 17% of jawbone primary cases, and to date, only a very small number of reports have been described in the literature [1-7]. In many instances, preoperative imaging, cytological evaluation, and biopsy often result in misdiagnosis as a benign tumor. This report presents a case of primary intraosseous mucoepidermoid carcinoma that was definitively diagnosed four years after the initial surgery. In this report, we describe a rare case of maxillary primary intraosseous mucoepidermoid carcinoma that was initially misdiagnosed, emphasizing the diagnostic challenges and utility of molecular testing.

## Case presentation

A 58-year-old woman with no history of smoking or alcohol consumption underwent surgery at the oral and maxillofacial surgery department of another hospital four years earlier to remove a left maxillary tumor. The pathological diagnosis at that time was a calcified epithelial odontogenic tumor. A cystic lesion in the left pterygoid region was also noted; however, the surgical team judged that intervention in this area would be challenging and opted for a “wait and see” approach. During follow-up, CT imaging revealed gradual growth of the residual tumor, leading to the patient’s referral to our department for further treatment.

The initial surgery for the left maxillary tumor performed in the previous hospital was conducted via an intraoral approach. The tumor was located in the left maxillary tuberosity. At that time, although lesions were also identified in the pterygoid region, they were not resected due to anatomical difficulties. The lesions in the maxilla and the pterygoid region were not connected.

**Figure 1:**
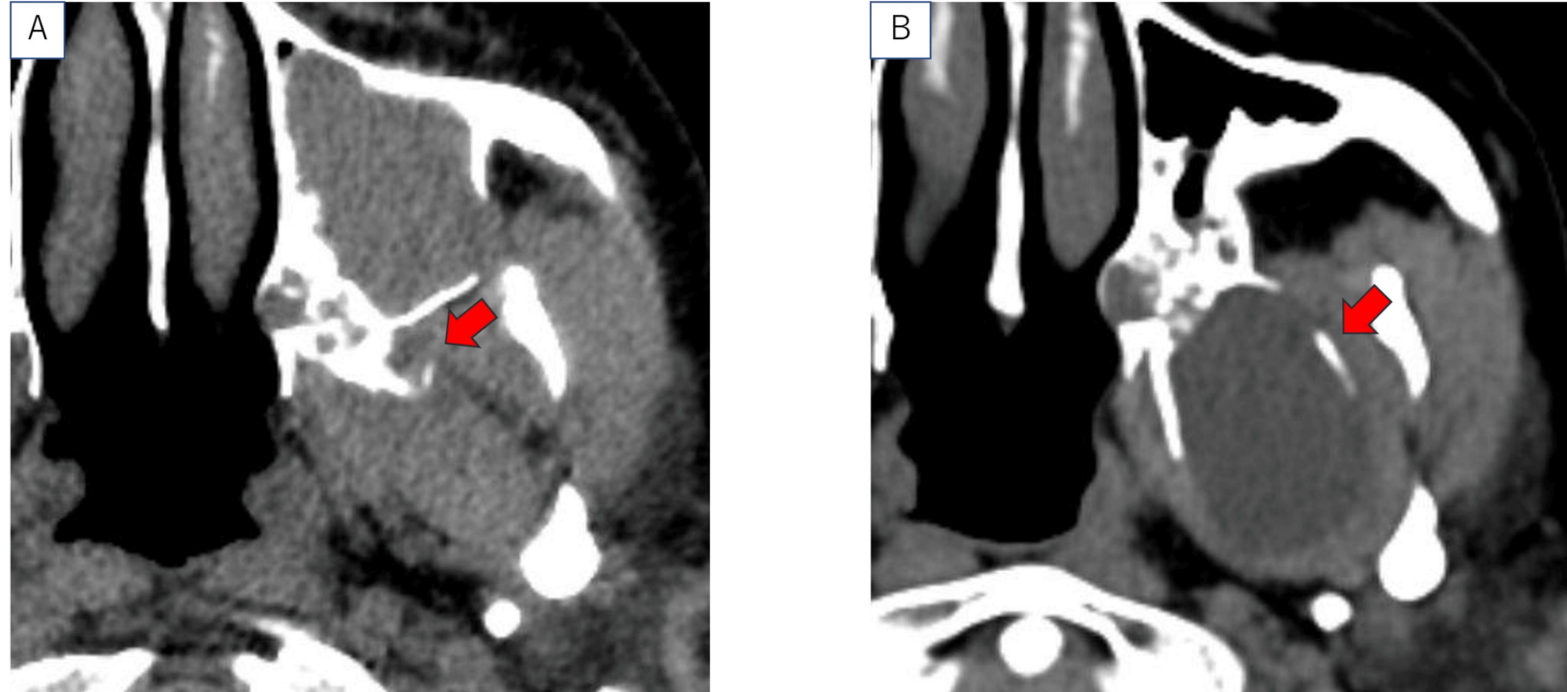
Horizontal cross-sectional CT image. Horizontal cross-sectional CT image taken at another hospital four years ago (A) and at our hospital during the first consultation (B). The red arrow indicates the lesion.

On presentation, no abnormalities were detected through nasal endoscopy. As shown in Figure 1A, the CT image from four years earlier demonstrated a soft tissue shadow measuring 1 cm in diameter on the left sphenoid wing. As shown in Figure 1B, it had increased to 4 cm by the time the patient visited our department.

**Figure 2:**
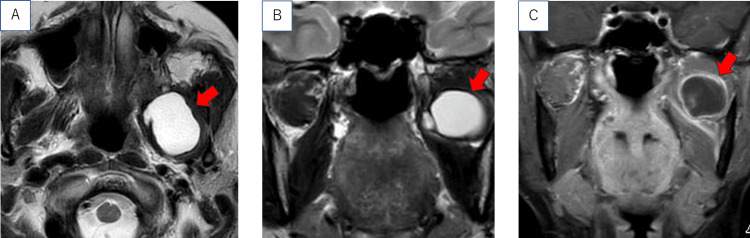
MRI images taken during the first consultation at our hospital. (A) T2-weighted horizontal section. (B) T2-weighted coronal section. (C) Contrast-enhanced T1-weighted coronal section. The red arrow indicates the lesion.

As shown in Figure 2, MRI examination demonstrated a cystic lesion measuring 4 cm in diameter, extending from the left maxilla to the infratemporal fossa. The tumor had progressed posteriorly, but there was no infiltration into the cranial nerves, and no symptoms were observed.

Since the lesion was located in an anatomically challenging area for outpatient biopsy and complete removal appeared feasible, we elected to perform both diagnosis and treatment in a single intervention using endoscopic transnasal surgery.

Under general anesthesia, we initially performed a nasal septoplasty through the right nasal cavity, the side opposite the tumor, to establish a wide working space in the left nasal cavity. Subsequently, an endoscopic modified medial maxillectomy (EMMM) was carried out to create a spacious corridor, allowing access to the posterior wall of the maxillary sinus. A pedicled mucosal flap was then constructed from the maxillary sinus mucosa, with the attachment at the sinus floor and the free edge extending as a long flap from the posterior to the superior wall of the maxillary sinus [8]. To secure an even wider surgical field, the natural ostium of the maxillary sinus was widely opened from the middle meatus. The posterior wall of the maxillary sinus was removed using a chisel and diamond burr, exposing the infratemporal fossa. While the maxillary artery and sphenopalatine artery were preserved, the descending palatine artery was cauterized and transected using a coblation device (Coblator II Surgery System™; ArthroCare, Sunnyvale, CA, USA), as it was inflowing into the lesion. The tumor was encased by bone tissue on all sides. The pterygoid process, including both its medial and lateral plates, was removed using the chisel and diamond burr. The medial and lateral pterygoid muscles were carefully dissected from the tumor using the Coblator™, and the tumor was excised en bloc. Finally, the resection site was covered with the pedunculated mucosal flap from the maxillary sinus, which had been prepared in advance, and the procedure was concluded.

**Figure 3:**
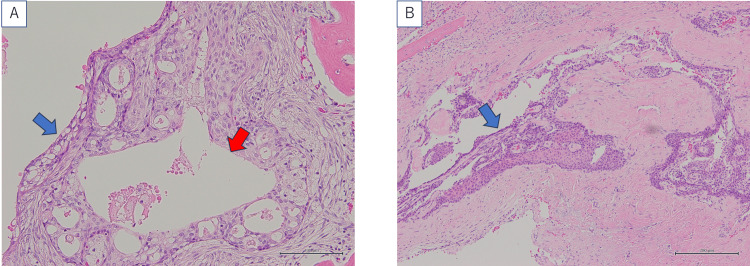
Histopathological finding of the removed tumor. (A) Cystic change (red arrow) and mucous cells (blue arrow). (B) Differentiation into non-keratinizing squamous epithelium (blue arrow). Calibration bars = 100 μm.

The postoperative pathological diagnosis was low-grade mucoepidermoid carcinoma. As shown in Figure 3, the tumor exhibited cystic degeneration and differentiation into non-keratinizing squamous epithelium.

**Figure 4:**
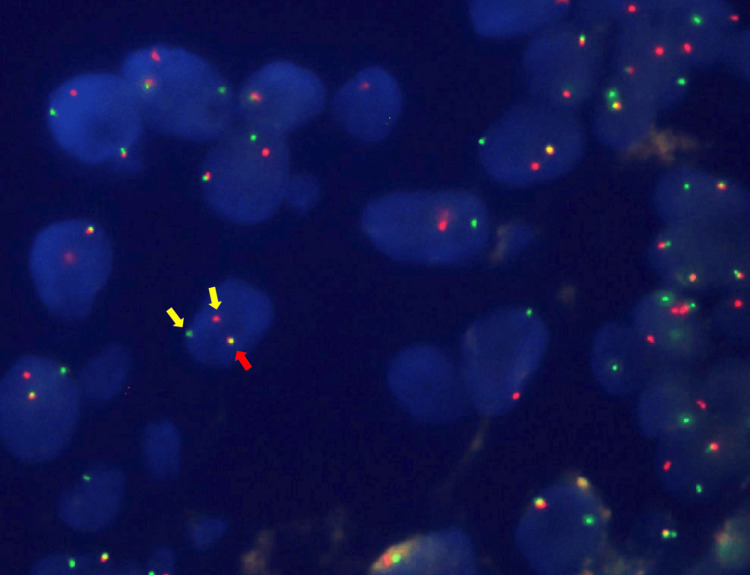
Fluorescence in situ hybridization of the MAML2 gene. Split positive (yellow arrow). Split negative (red arrow).

Small glandular cavities and a few goblet cells were observed in some areas. No mitotic figures were detected. Although an enamel epithelial tumor was considered as a differential diagnosis, it was excluded due to the presence of mucous cells. As shown in Figure 4, a split was observed in the fluorescence in situ hybridization (FISH) test of the *MAML2* gene, and, in conjunction with the morphological findings, the diagnosis of low-grade mucoepidermoid carcinoma was confirmed. The probe used in this time was the *MAML2* break-apart probe (ZytoVision, Bremerhaven, Germany).

To determine the relationship with the adjacent maxillary tumor that had occurred four years ago, we ordered the pathological specimens from the previous hospital and applied a FISH test on the *MAML2 *gene. The results revealed that the tumor removed four years ago was not a calcified epithelial odontogenic tumor but a mucoepidermoid carcinoma, indicating that the present tumor was a remnant of the maxillary tumor treated four years ago. Postoperative irradiation was planned due to the close margins of the tumor resection. Imaging examination for metastatic lesions did not reveal any lesions suggestive of lymph node metastasis and distant metastasis from the mucoepidermoid carcinoma. Although renal cell carcinoma was incidentally diagnosed and treatment for the renal tumor was prioritized, postoperative irradiation for the mucoepidermoid carcinoma was not performed. The patient experienced a transient limitation of mouth opening after surgery, but this condition gradually improved during follow-up. Two and a half years have passed since the surgery, with no evidence of tumor recurrence or metastasis.

## Discussion

Previous reports on the diagnosis of primary intraosseous mucoepidermoid carcinoma of the jaw have identified the following diagnostic criteria: (1) radiographic evidence of bone destruction with normal cortical bone, (2) positive mucicarmine staining in histopathological examination, and (3) the absence of any other primary squamous cell carcinoma [9]. Additionally, it is essential to rule out the possibility of the tumor arising from surrounding tissues of the jawbone, including the oral cavity, nasal cavity, maxillary sinus mucosa, salivary glands, and skin, and to exclude the possibility of metastasis from other parts of the body. Mucicarmine staining is a test used to identify epithelial mucus. In this case, hematoxylin-eosin staining revealed clear mucus-producing cells; therefore, mucicarmine staining was not performed. The diagnosis of low-grade mucoepidermoid carcinoma was confirmed with the presence of a split in the FISH test. The present case met diagnostic criteria (1) and (3), leading to the conclusion of primary intraosseous mucoepidermoid carcinoma.

An intraosseous mucoepidermoid carcinoma often presents as a unilocular or multilocular cystic-like lesion on imaging studies. As it grows slowly and does not cause bone destruction, even in the presence of bulging or defects in the cortical bone, it may be misdiagnosed as a benign tumor, such as an enamel epithelial tumor. A previous report demonstrated that only five out of 22 cases (22%) were diagnosed as malignant tumors prior to surgery. Attention must be given to cases where preoperative biopsy is challenging due to anatomical considerations and where benign tumors are suspected based on imaging findings, as in the present case. Additionally, fine-needle aspiration or biopsy of the tumor has been reported to be misdiagnosed as a benign tumor [10]. The histopathological features of intraosseous mucoepidermoid carcinoma and odontogenic cysts are similar, which complicates the preoperative diagnosis.

The first choice of treatment for primary intraosseous mucoepidermoid carcinoma of the jawbone is surgical resection. While the recurrence rate for enucleation and curettage has been reported to be 40%, it is only 4% when resection is performed with a sufficient safety margin [11]. A complete cure with radiation therapy alone is challenging, as the tumor's sensitivity to radiation is low. Postoperative radiation therapy is indicated only for cases with high malignancy, positive resection margins, or positive cervical lymph node metastasis [10]. It is essential to perform a resection that ensures a pathological safety margin. Due to the difficulty of preoperative diagnosis, planning in advance is challenging, and in some cases, radiation therapy may be required after surgery.

In mucoepidermoid carcinoma, reports have linked fusion genes caused by gene mutations, which serve as an aid in diagnosing difficult cases of the disease. Previous studies have shown that the *CRTC1-MAML2 *fusion gene is positive in 38% of cases, and the *CRTC3-MAML2* fusion gene is positive in 6% of cases [12,13]. Since these tests require reverse transcription PCR, frozen pathological tissue samples are needed. Although formalin-fixed paraffin-embedded tissue samples can also be used, they require a larger amount of tissue for testing. The utility of confirming the *MAML2* gene split using paraffin sections and FISH has also been reported. This method is more practical than reverse transcription PCR, as it can be performed using commercially available probes. Although the number of facilities capable of performing this test is limited, a diagnosis can be made within a few days. In a previous study of 95 cases of mucoepidermoid carcinoma, it was reported that the* MAML2* split was positive in 62 cases, while the *CRTC1-MAML2* fusion gene was positive in 37 cases, and the *CRTC3-MAML2* fusion gene was positive in six cases by reverse transcription PCR; the combined sensitivity and specificity of these tests were 75% and 100%, respectively [14,15].

Therefore, the *MAML2* split is an extremely useful auxiliary diagnostic tool that improves the positive diagnosis rate of mucoepidermoid carcinoma. In this challenging case, we were able to diagnose the patient with mucoepidermoid carcinoma based on the *MAML2* split identified in the FISH test. Furthermore, when the surgical specimen from another hospital, obtained four years ago, was re-examined using the FISH test, it was confirmed to be mucoepidermoid carcinoma. In this case, the patient had been under long-term observation for four years, despite the fact that the tumor was malignant due to the misdiagnosis of the initial pathological diagnosis at the previous hospital. To prevent similar issues in the future, it is essential to quickly reach the correct diagnosis while considering intraosseous mucoepidermoid carcinoma as a differential diagnosis.

## Conclusions

We reported a case of primary intraosseous mucoepidermoid carcinoma of the maxilla that was correctly diagnosed four years after the initial surgery. Due to its extreme rarity, diagnosis based solely on imaging and histopathological examinations can be challenging. Therefore, to reach an accurate diagnosis, it is essential to consider this disease as a differential diagnosis and to be familiar with the appropriate diagnostic methods. In particular, *MAML2* FISH testing proved to be a crucial tool in achieving an accurate diagnosis. It could become a useful tool in similar cases in the future.
